# Continuation of Exercise Is Necessary to Inhibit High Fat Diet-Induced β-Amyloid Deposition and Memory Deficit in Amyloid Precursor Protein Transgenic Mice

**DOI:** 10.1371/journal.pone.0072796

**Published:** 2013-09-04

**Authors:** Masato Maesako, Kengo Uemura, Ayana Iwata, Masakazu Kubota, Kiwamu Watanabe, Maiko Uemura, Yasuha Noda, Megumi Asada-Utsugi, Takeshi Kihara, Ryosuke Takahashi, Shun Shimohama, Ayae Kinoshita

**Affiliations:** 1 School of Human Health Sciences, Kyoto University Graduate School of Medicine, Kyoto, Japan; 2 Department of Neurology, Kyoto University Graduate School of Medicine, Kyoto, Japan; 3 Ishiki Hospital, Kagoshima, Japan; 4 Department of Neurology, Sapporo Medical University, Sapporo, Japan; National Institute on Aging Intramural Research Program, United States of America

## Abstract

High fat diet (HFD) is prevalent in many modern societies and HFD-induced metabolic condition is a growing concern worldwide. It has been previously reported that HFD clearly worsens cognitive function in amyloid precursor protein (APP) transgenic mice. On the other hand, we have demonstrated that voluntary exercise in an enriched environment is an effective intervention to rescue HFD-induced β-amyloid (Aβ) deposition and memory deficit. However, it had been unclear whether consumption of HFD after exercising abolished the beneficial effect of exercise on the inhibition of Alzheimer's disease (AD) pathology. To examine this question, we exposed wild type (WT) and APP mice fed with HFD to exercise conditions at different time periods. In our previous experiment, we gave HFD to mice for 20 weeks and subjected them to exercise during weeks 10–20. In the present study, mice were subjected to exercise conditions during weeks 0–10 or weeks 5–15 while being on HFD. Interestingly, we found that the effect of exercise during weeks 0–10 or weeks 5–15 on memory function was not abolished in WT mice even if they kept having HFD after finishing exercise. However, in APP transgenic mice, HFD clearly disrupted the effect of exercise during weeks 0–10 or weeks 5–15 on memory function. Importantly, we observed that the level of Aβ oligomer was significantly elevated in the APP mice that exercised during weeks 0–10: this might have been caused by the up-regulation of Aβ production. These results provide solid evidence that continuation of exercise is necessary to rescue HFD-induced aggravation of cognitive decline in the pathological setting of AD.

## Introduction

Alzheimer's disease (AD), the occurrence of which is largely sporadic, is characterized by deficits in memory and other cognitive functions. Amyloid plaque is one of the pathological hallmarks of AD and it is composed of β-amyloid (Aβ). Aβ is derived from the amyloid precursor protein (APP) via proteolytic cleavages by β- and γ-secretases. Aβ, in turn, is degraded by several Aβ-degrading enzymes including neprilysin or insulin-degrading enzyme. A widely accepted hypothesis about AD pathogenesis is the amyloid cascade hypothesis, in which Aβ plays a crucial role as an upstream molecule in neurodegeneration [1]. Considering that Aβ accumulation is clearly observed almost a decade before cognitive impairment is seen in AD patients [2], [3], there is growing consensus that prevention of AD at the earliest stage is desirable.

Metabolic conditions including obesity and diabetes mellitus are becoming severe problems. In modern society, the spread of high caloric diet including high fat diet (HFD) may be the critical cause of metabolic abnormalities. Of course, metabolic conditions are known risk factors of vascular dementia but they could also risk factors of sporadic AD [4]–[6]. It has been consistently seen that feeding HFD to APP transgenic mice worsens the pathological alterations of Aβ metabolism and memory impairment [7]. On the other hand, the paradigm of environmental enrichment has been frequently used in experiments wherein mice can conduct exercise voluntarily in a larger cage, with complex stimuli (e.g., running wheels, toys), thus being provided with more physical and intellectual stimulation than mice housed in standard conditions [8], [9]. Recently, we set up an enriched condition focused on physical stimulation and demonstrated that voluntary exercise inhibits HFD-induced Aβ deposition and memory deficit in APP transgenic mice [10]. More importantly, we have shown that voluntary exercise is more effective than diet control in our experimental setting [11]. Consistent with our results, many reports support the theory that physical activity is closely associated with a decreasing risk of cognitive impairment and that exercise is an effective strategy to prevent development of AD [12]–[15].

Considering that metabolic condition is a growing issue worldwide, we wondered whether continuing to have HFD after exercising would abolish the beneficial effect of exercise on memory function. In the present study, we demonstrated that the effect of exercise on memory function was not abolished in WT mice even if they kept having HFD after finishing exercise. However, the exercise-induced improvement of memory was clearly disrupted in APP transgenic mice. We showed that consumption of HFD after exercising increased the level of Aβ oligomer in APP transgenic mice. Besides, we speculated that the development of Aβpathology might have been caused by increase in Aβproduction due to HFD. The results indicated that HFD after finishing exercise might immediately worsen AD pathology, and later disrupt the effect of exercise on memory function. Therefore, continuation of exercise is necessary to inhibit HFD-induced cognitive impairment in the pathological setting of AD.

## Methods

### Ethics statement

All animal experiments in this study were performed with the approval of the Animal Experiment Committees of Kyoto University, Graduate School of Medicine (Permit Number: 09597). All experiments were conducted in strict adherence to the relevant international guidelines. Every effort was made to minimize suffering of the animals.

### Animals, dietary and exercise conditions

WT (C57BL/6 J) mice were obtained from Charles River Laboratories Japan, Inc. (Japan). Human APP transgenic mice (J20 mice) overexpressing familial AD-linked mutations bearing both Swedish (K670N/M671L) and Indiana (V717F) mutation were imported from the Jackson Laboratory (USA) [16]. They were maintained as heterozygotes. Since HFD worsened memory deficit and Aβ deposition in female APP transgenic mice better than in male (unpublished observation), female mice were used in these experiments. To establish APP transgenic mice fed with HFD (APP-HFD mice), age matched female were exposed to an established HFD (caloric composition, 60% fat, 20% carbohydrate, and 20% protein, Research Diet, Inc., Canada) for 20 weeks, from 2–3 to 7–8 months of age. As a control diet, female APP transgenic mice were exposed to a standard diet (10% fat, 70% carbohydrate, and 20% protein, Oriental Yeast Co., Ltd., Japan). In voluntary exercise (Ex) condition, a cage was changed to an enriched environment which was a 2.4 times larger than the standard cage, and was equipped with a running wheel (12 cm in diameter), toys and a stand. To examine the effects of exercise at different periods on APP-HFD mice, female APP-HFD mice spent weeks 0–10 (APP-HFD+Ex 0–10 mice, n = 8) or weeks 5–15 (APP-HFD+Ex 5–15 mice, n = 7) in exercise conditions in the presence of HFD. As a control, we used female APP-HFD mice which spent weeks 10–20 (APP-HFD+Ex 10–20 mice, n = 6) in exercise conditions which we had previously established [10]. In the experiments of metabolic analyses and memory test, we examined the effect of exercise at different periods on WT mice fed HFD (WT-HFD mice), using the same paradigm (n = 5, each group). In order to examine the running length per day, we monitored the number of running wheel rotations and estimated it as follows:

*n* (the number of running wheel rotations) ×*12* (cm/diameter) ×*3.14*.

After dietary and motile manipulations, metabolic changes in these mice were analyzed, which was followed by assessment of memory function through Morris water maze test, as described below. After the analysis of memory function, brains were extracted and cut sagitally into left and right hemispheres. Tribromoethanol was used for anesthesia in the surgical procedures. The left hemisphere was fixed in 4% paraformaldehyde for histological analysis. After removing the olfactory lobe and the cerebellum, the right hemisphere was rapidly frozen in liquid nitrogen for biochemical analysis.

### Assessment of metabolic changes

Blood was collected from the tail-vein. To assess glucose intolerance in these mice, we conducted intra-peritoneal glucose tolerance test (IGTT). Mice were given a single intra-peritoneal injection of glucose (2 g/kg body weight) after 14 hours fasting, and blood was collected periodically over 2 hours (fasting, 30 min, 60 min and 120 min). Plasma glucose content was measured by using LabAssay Glucose (Wako, Japan). Plasma insulin concentration was measured by enzyme-linked immunosorbent assay (ELISA) kit specific to insulin (Morinaga Seikagaku, Japan).

### Morris water maze test

In order to assess spatial navigation learning and memory retention, Morris water maze test was conducted using a pool of water (diameter 120 cm, height 25 cm, temperature 21±1°C).

#### Visual cue phase

Visible platform training was performed to measure the motivation and swimming speed of mice to find a platform. Distal cues were removed from around the pool, and the platform was labeled with a flag and placed 1 cm above the surface of the water in the center of a quadrant. Mice were placed in the maze and allowed to explore the maze for 60 sec; if they reached the visible platform, they were allowed to remain there for 20 sec before being returned to their cages. If they did not find the platform within 60 sec, the experimenter led them to the platform and let them remain there for 20 sec. The training was completed once each animal received six trials. This training was performed for 1 day.

#### Acquisition phase

We measured the ability of the mice to understand the spatial relationship between a safe, but invisible platform of 10 cm diameter (submerged 1 cm below water level), and visual cues surrounding the maze. The platform was located in the center of one of the four quadrants, and several extramaze cues were distributed across the walls surrounding the pool. During the acquisition phase of training, each mouse received four daily hidden platform training trials with 12 min intervals for 5 consecutive days. The animals were allowed 60 sec to locate the platform and 20 sec to rest on it. The mice that failed to find the platform were led there by the experimenter and allowed to rest there for 20 sec.

#### Probe trial phase

A day after the last acquisition trial, a single 60 sec probe trial was administered to assess spatial memory retention. For the probe trial, the animals were returned to the pool without the platform present, and the parameters were recorded to assess the ability of the mouse to remember the previous location of the platform.

Performance was recorded with an automated tracking system (ANY-maze, Brain Science. Idea. Co., Ltd., Japan) during all the phases of training. During the visual cue phase of training, the speeds at which the mice reached to the platform were used to compare the activity of the performance among each group. During the acquisition phase, time to goal (latency to reach the platform) was subsequently used to analyze and compare the performance between the different treatment groups. The time taken to get to the position where platform presented and the time spent in goal quadrant were analyzed during the probe trials.

### Measurement of Aβ by ELISA

In order to prepare the samples for detection of Aβ, mice cerebrums were homogenized with Tris buffer saline (TBS) with protease inhibitor cocktail (Roche, Germany). The homogenate was centrifuged at 100,000 g for 1 hour, and the supernatant was collected as the TBS-extracted fraction. The pellet was washed in TBS and centrifuged at 100,000 g for 1 hour. Seventy percent formic acid (FA) was added to the pellet, which was homogenized again. The homogenate was incubated for 1 hour at 4°C and then centrifuged at 100,000 g for 1 hour at 4°C. The resultant supernatant was collected as the FA-extracted fraction, which was neutralized with a 20-fold volume of 1 M Tris buffer (pH 11.0).

The levels of Aβ 40 and Aβ 42 in FA fraction, or Aβ oligomer in TBS fraction were measured using ELISA kits specific to Aβ 40, Aβ 42, or Aβ oligomer (82E1-specific) (Immuno-Biological Laboratories Co., Ltd., Japan), and in accordance with the manufacturer's instructions. We used a standard format for measuring monomeric Aβ species with the use of C-terminal capturing antibodies and N-terminal or mid-region detecting antibodies. To detect Aβ oligomer species, the same N-terminal antibody, 82E1 (to Aβ residues 1–16, Immuno-Biological Laboratories, Inc, USA), was used for both capture and detection.

### Immunoblotting and filter trap assay

For immunoblotting analysis, mice cerebrums were extracted in radio-immunoprecipitation assay (RIPA) buffer (50 mM Tris-HCl, 150 mM NaCl, 1% Triton X100, 1% NP-40, 0.5% Deoxycholate, 0.1% SDS, pH 8.0) with protease inhibitor cocktail and sufficiently homogenized on ice. The samples were incubated for one night at 4°C and centrifuged at 14,000 g for 20 min. The supernatants were directly used for Western blot analysis. The detailed protocol has been described previously [10], [11]. In the previous experiments, we had used two types of gels (5–20% polyacrylamide gradient gels (Atto, Japan) and 4–12% NuPAGE Bis-Tris gel (Invitrogen, USA)) and two types of antibodies (rabbit polyclonal anti-APP C-terminal antibody and mouse monoclonal 6E10 antibody). Since the experiment using 4–12% NuPAGE Bis-Tris gel and rabbit polyclonal anti-APP C-terminal antibody was the most quantitative, the same as used in the present study. Rabbit polyclonal anti-APP C-terminal antibody was obtained from SIGMA.

Filter trap assay was conducted as described previously [11]. Briefly, the protein concentration of the cerebrum samples in TBS-extracted fraction was measured and an equal amount of protein was subjected to vacuum filtration through a 96-well dot blot apparatus (Bio-Rad Laboratories, USA) containing 200 nm pore-sized nitrocellulose membrane. The membrane was then incubated with the primary antibody at 4°C overnight. It was then blocked by TBS containing 4% skimed milk, and incubated with HRP-linked secondary antibody (GE Healthcare, UK; diluted 1∶2000) for 1 hour. Next, it was developed using the ECL Western Blotting Analysis System (GE Healthcare). Anti-oligomer antibody (A11, Invitrogen) was used for the detection of Aβ oligomer in the TBS soluble fraction.

### Immunohistochemistry

The paraformaldehyde-fixed and paraffin-embedded tissue sections of mice were deparaffinized and rehydrated. The antigens of tissue sections were activated by autoclave for 10 minutes in 10 mM citrate buffer (pH 6.0). Endogenous peroxidase activity was suppressed by 0.6% hydrogen peroxide. Thereafter, tissue sections were blocked by horse serum and incubated with primary antibodies for 1 hour. The sections were then incubated with Histofine Simple Stain MAX PO (Nichirei Corporation, Japan) for 1 hour. Subsequently, the labeling was visualized by incubation with a solution of 3,3-diaminobenzidine (Merck & Co., Inc, USA) with hydrogen peroxide. All images were visually analyzed using a microscope, ECLIPSE 80i (Nikon Corporation). Anti-Aβ (6E10) antibody (1∶200; SIGMA) was used for the detection of Aβ plaque.

### Neprilysin activity assay

The proteolytic activity of neprilysin was measured as described previously but with minor modifications [17]. Briefly, mice cerebrums were extracted in RIPA buffer and protein concentrations were analyzed. 25 μg of extracted samples were incubated with 50 μM substrate3-dansyl-D-Ala-Gly-p-(nitro)-Phe-Gly (DAGNPG) (SIGMA) and 1 μM captopril- angiotensin converting enzyme (ACE) inhibitor- in 200 μl of 50 mM Tris-HCl buffer (pH 7.6) for 1 hour at 37°C. Reactions were stopped by heating samples to 100°C for 5 min, followed by 5,000 g×5 min centrifugation. The 180 μl of supernatant was diluted into 400 μl of 50 mM Tris-HCl buffer (pH 7.6) and fluorescence was determined using Infinite 200 PRO (Tecan Japan Co., Ltd., Japan) (excitation 342 nm, emission 562 nm).

### Statistical analysis

All values are given in means ± SE. Comparisons were performed using an unpaired Student's t-test. For comparison of multiparametric analysis, we used one-way factorial ANOVA, followed by a post-hoc analysis using Bonferroni post-hoc test. Statistical significance of differences between mean scores during IGTT and acquisition phase of Morris test were assessed with two-way repeated-measures ANOVA (general linear model/RM-ANOVA) and Bonferroni post-hoc analysis for multiple comparisons. The value p<0.05 was considered to indicate a significant difference.

## Results

### Effects of exercise at different periods on the metabolic conditions in APP-HFD mice

**Figure 1:**
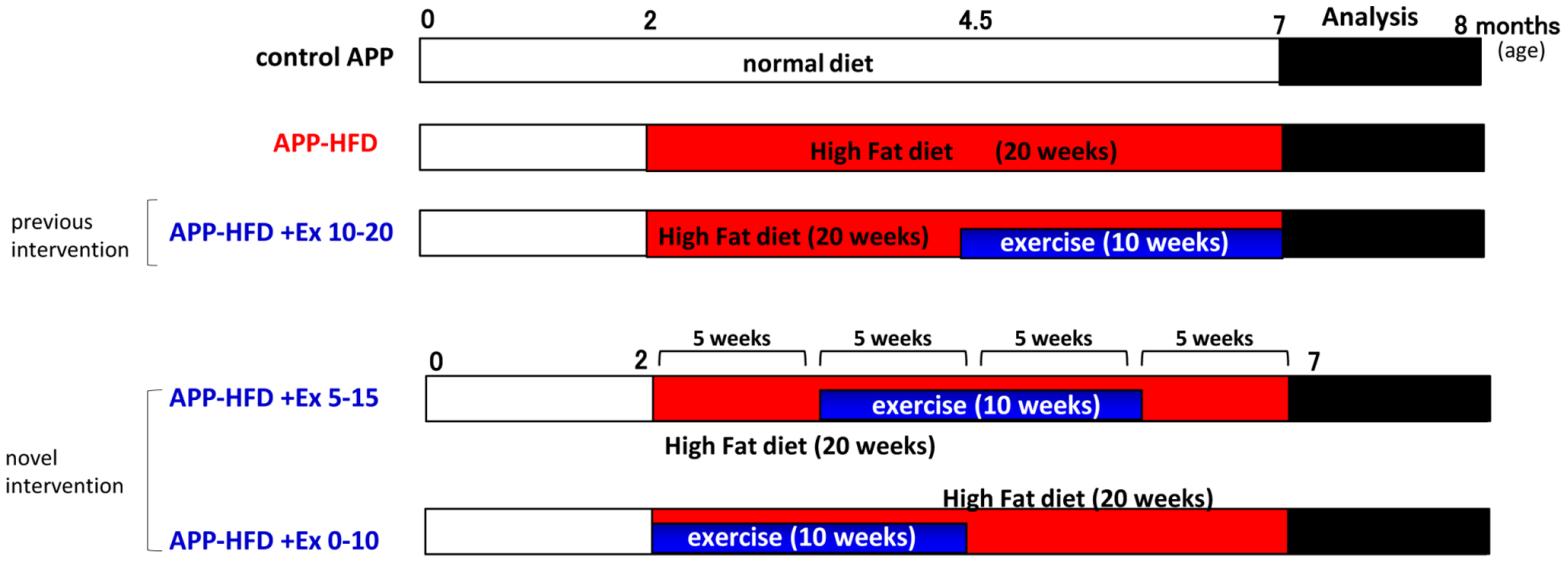
Exercise treatment given to APP-HFD mice at different time periods. Schematic presentation of the exercise treatment given to APP-HFD mice. Female APP transgenic mice were maintained on standard diet in standard laboratory cages until they were 2–3 months old. Then, the mice were separated into 5 groups. In the control group, the mice were fed with a standard diet in standard laboratory cages for 20 weeks (control APP mice) (top row, n = 5). In the HFD-induced group, the mice were fed HFD in standard laboratory cages for 20 weeks (APP-HFD mice) (2^nd^ row, n = 5). In the exercise-induced group during weeks 10 to 20 of HFD, the mice spent 10 weeks in standard laboratory cages, and then spent 10 weeks in enrichment cages in the presence of HFD (APP-HFD+Ex 10–20 mice) (3^rd^ row, n = 6). As novel interventions, in the exercise-induced group during 5 to 15 weeks of HFD, the mice spent 5 weeks in standard laboratory cages, 10 weeks in enrichment cages, and then 5 weeks in standard laboratory cages in the presence of HFD (APP-HFD+Ex 5–15 mice) (4^th^ row, n = 7). In the exercise-induced group on 0 to 10 weeks of HFD, the mice spent 10 weeks in enrichment cages, and then spent 10 weeks in standard laboratory cages in the presence of HFD (APP-HFD+Ex 0–10 mice) (5^th^ row, n = 8). After 20 weeks, metabolic conditions of these mice were analyzed, followed by ethological, histochemical and biochemical analyses targeting AD pathophysiology.

**Figure 2:**
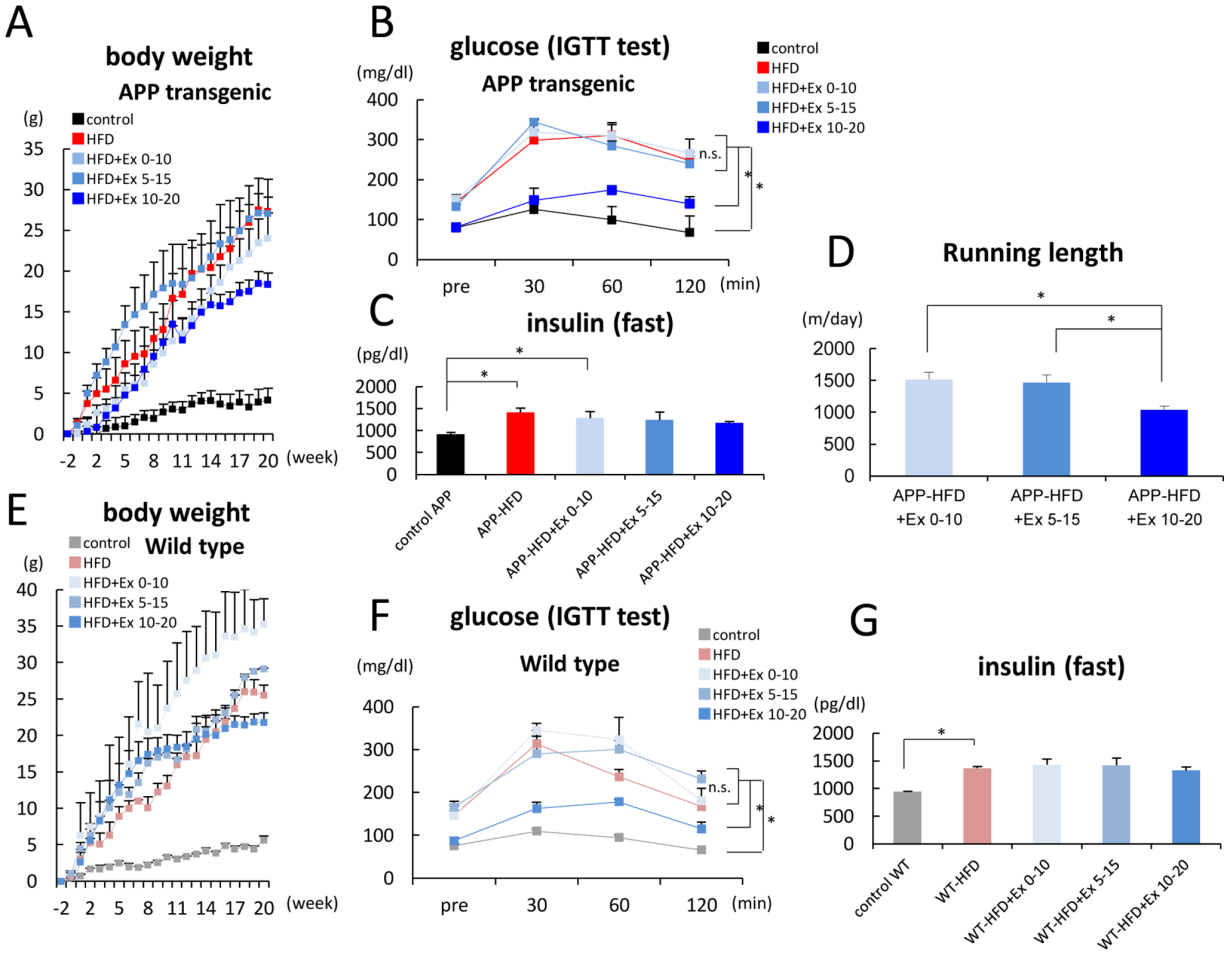
HFD after finishing exercise deteriorated glucose tolerance in APP-HFD mice. (*A*) Relative body weight changes over 20 weeks in control APP, APP-HFD, APP-HFD+Ex 0–10, APP-HFD+Ex 5–15 and APP-HFD+Ex 10–20 mice. The body weight 2 weeks before each diet was regarded as the baseline (0 g). (*B*) Blood glucose levels during glucose tolerance test after an intra-peritoneal injection of glucose (2 g/kg body weight). Fasting glucose levels in APP-HFD+Ex 0–10 mice (F _(4, 20)_  = 9.03, p<0.001) and in APP-HFD+Ex 5–15 mice (p = 0.006) were higher than those in APP-HFD+Ex 10–20 mice. Glucose tolerance abilities in APP-HFD+Ex 0–10 mice (F _(4, 60)_  = 16.17, p<0.001) and in APP-HFD+Ex 5–15 mice (p<0.001) were worse than those in APP-HFD+Ex 10–20 mice. The glucose tolerance in APP-HFD mice was the same as those in APP-HFD+Ex 0–10 mice and APP-HFD+Ex 5–15 mice. n.s. indicated not significant. * indicated p<0.05. (*C*) Blood insulin levels during fasting. Plasma insulin levels in APP-HFD+Ex 0–10 mice and in APP-HFD+Ex 5–15 mice were not different from those in APP-HFD+Ex 10–20 mice (F _(4, 20)_  = 2.22). * indicated p<0.05. (*D*) Average running distance using a running wheel per day (m/day). Running distance was estimated from the number of running wheel rotations. The lengths in APP-HFD+Ex 0–10 mice (F _(2, 12)_  = 7.61, p = 0.003) and in APP-HFD+Ex 5–15 mice (p = 0.008) were significantly longer than those in APP-HFD+Ex 10–20 mice. * indicated p<0.05. (*E*) Relative body weight changes over 20 weeks in control WT, WT-HFD, WT-HFD+Ex 0–10, WT-HFD+Ex 5–15 and WT-HFD+Ex 10–20 mice. The body weight 2 weeks before each diet was regarded as the baseline (0 g). (*F*) Blood glucose levels during glucose tolerance test after an intra-peritoneal injection of glucose (2 g/kg body weight). Fasting glucose levels in WT-HFD+Ex 0–10 mice (F _(4, 10)_  = 12.72, p = 0.006) and in WT-HFD+Ex 5–15 mice (p<0.001) were higher than that in WT-HFD+Ex 10–20 mice. Glucose tolerance abilities in WT-HFD+Ex 0–10 mice (F _(4, 30)_  = 29.98, p<0.001) and in WT-HFD+Ex 5–15 mice (p<0.001) were lower than that in WT-HFD+Ex 10–20 mice. n.s. indicated not significant. * indicated p<0.05. (*G*) Insulin levels during fasting. Insulin levels in WT-HFD+Ex 0–10 mice and in WT-HFD+Ex 5–15 mice were not different from that in WT-HFD+Ex 10–20 mice (F _(4, 10)_  = 7.24).

In our previous experiment, we fed APP transgenic mice with HFD for 20 weeks and the mice were subjected to exercise conditions during weeks 10–20 [10]. However, in the present study, we exposed APP-HFD mice to exercise conditions at different periods: weeks 0–10 (APP-HFD+Ex 0–10 mice) and weeks 5–15 (APP-HFD+Ex 5–15 mice) (Figure 1). The increase of body weight was suppressed during exercising in APP-HFD+Ex 0–10 mice and APP-HFD+Ex 5–15 mice, although it was not as evident as that in APP-HFD+Ex 10–20 mice (Figure 2A). The food intake of APP-HFD+Ex 10–20 mice was the same or mildly increased compared with that of APP-HFD+Ex 0–10 mice and APP-HFD+Ex 5–15 mice (Figure S1), indicating that the attenuation of body weight in APP-HFD+Ex 10–20 mice was not caused by reduction of food intake. Fasting glucose levels in APP-HFD+Ex 0–10 mice and APP-HFD+Ex 5–15 mice were not different from those in APP-HFD mice, which were higher than that in APP-HFD+Ex 10–20 mice (Figure 2B. pre). Glucose tolerance ability was clearly deteriorated in APP-HFD+Ex 0–10 mice and APP-HFD+Ex 5–15 mice compared with that in APP-HFD+Ex 10–20 mice (Figure 2B). Insulin levels were not different among APP-HFD mice, APP-HFD+Ex 0–10 mice, APP-HFD+Ex 5–15 mice and APP-HFD+Ex 10–20 mice (Figure 2C). Importantly, running distances in APP-HFD+Ex 0–10 mice and APP-HFD+Ex 5–15 mice were longer than those in APP-HFD+Ex 10–20 mice (Figure 2D), indicating that the deterioration of glucose metabolism in APP-HFD+Ex 0–10 mice and APP-HFD+Ex 5–15 mice was not caused by reduction of physical activity. We also examined HFD-induced metabolic changes in WT mice that were subjected to exercise. As in APP transgenic mice, HFD after finishing exercise increased body weight (Figure 2E) and reduced glucose tolerance (Figure 2F and G) in WT-HFD+Ex 0–10 mice and WT-HFD+Ex 5–15 mice. Collectively, these results indicated that HFD after exercising might abolish the effects of exercise in maintaining reduced body weight and improving glucose intolerance.

### Effect of exercise on memory function was not abolished by HFD in WT but in APP transgenic mice

**Figure 3:**
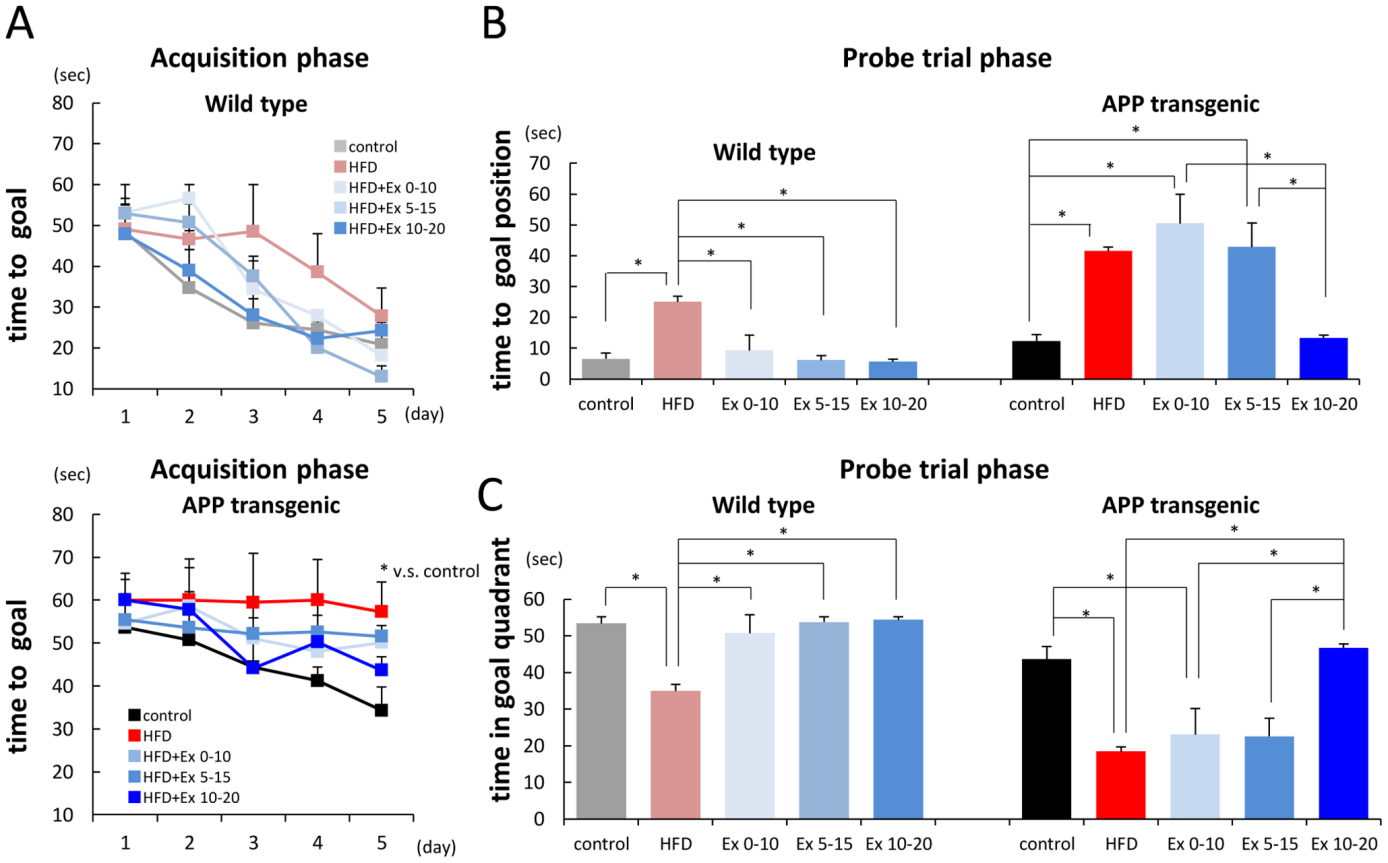
Effect of exercise on memory function was abolished by HFD in APP transgenic mice. (*A*) The time to get to goal platform of exercise-treated WT-HFD mice (upper) and APP-HFD mice (lower) in the acquisition phase of Morris water maze test, 20 weeks after having HFD. WT-HFD+Ex 0–10 mice and WT-HFD+Ex 5–15 mice took the same time to get to the platform as WT-HFD+Ex 10–20 mice. Also, APP-HFD+Ex 0–10 mice and APP-HFD+Ex 5–15 mice tended to take longer than APP-HFD+Ex 10–20 mice to get to the platform; however, this was statistically insignificant. (*B*) Time taken to get to goal position of exercise-treated WT-HFD mice (left) and APP-HFD mice (right) in the probe trial phase of Morris water maze test, 20 weeks after having HFD. WT-HFD+Ex 0–10 mice (F _(4, 10)_  = 18.63, p<0.001) and WT-HFD+Ex 5–15 mice (p<0.001) took less time to reach the platform position than WT-HFD mice, but they took the same time as WT-HFD+Ex 10–20 mice. On the other hand, APP-HFD+Ex 0–10 mice (F _(4, 20)_  = 7.89, p<0.001) and APP-HFD+Ex 5–15 mice (p = 0.006) took longer to get to platform position than APP-HFD+Ex 10–20 mice. The time taken by APP-HFD+Ex 0–10 mice and APP-HFD+Ex 5–15 mice was the same as that by APP-HFD mice. * indicated p<0.05. (*C*) Time in goal quadrant of exercise-treated WT-HFD mice (left) and APP-HFD mice (right) in the probe trial phase of Morris water maze test, 20 weeks after having HFD. WT-HFD+Ex 0–10 mice (F _(4, 10)_  = 46.97, p<0.001) and WT–HFD+Ex 5–15 mice (p<0.001) spent more time in goal quadrant than WT-HFD mice, but spent the same time in goal quadrant as WT-HFD+Ex 10–20 mice. On the other hand, APP-HFD+Ex 0–10 mice (F _(4, 20)_  = 7.09, p = 0.003) and APP-HFD+Ex 5–15 mice (p = 0.003) spent less time in goal quadrant than APP-HFD+Ex 10–20 mice. The time for APP-HFD+Ex 0–10 mice and APP-HFD+Ex 5–15 mice was the same as that for APP-HFD mice. * indicated p<0.05.

To examine whether HFD after exercising abolished the positive effect of exercise on memory function, we conducted Morris water maze test on the mice 20 weeks after having HFD. During the acquisition phase, the acquisition time was equally improved in WT-HFD+Ex 0–10 mice, WT-HFD+Ex 5–15 mice and WT-HFD+Ex 10–20 mice (Figure 3A, upper). In the probe trial phase, the time to get to goal position in WT-HFD+Ex 0–10 mice and that in WT-HFD+Ex 5–15 mice was the same as that in WT-HFD+Ex 10–20 mice, but it was shorter than that in WT-HFD mice (Figure 3B, left). Also, the time in goal quadrant in WT-HFD+Ex 0–10 mice and that in WT-HFD+Ex 5–15 mice was the same as that in WT-HFD+Ex 10–20 mice, but it was longer than that in WT-HFD mice (Figure 3C, left). These results suggested that WT mice might remain the effect of exercise on memory function even if they kept having HFD after exercising. In contrast, the time to get to goal position in APP-HFD+Ex 0–10 mice and that in APP-HFD+Ex 5–15 mice was significantly longer than that in APP-HFD+Ex 10–20 mice, but it was the same as that in APP-HFD mice (Figure 3B, right). Besides, the time in goal quadrant in APP-HFD+Ex 0–10 mice and that in APP-HFD+Ex 5–15 mice was shorter than that in APP-HFD+Ex 10–20 mice, but it was the same as that in APP-HFD mice (Figure 3C, right). Notably, locomotor activities were not affected by different periods of exercise as exhibited by swimming speeds (Figure S2). Additionally, to examine whether exercise during weeks 0–10 and 5–15 actually improved memory function, we randomly selected two mice from each group- APP-HFD+Ex 0–10 mice, APP-HFD+Ex 5–15 mice and APP-HFD+Ex 10–20 mice- and examined their memory function at the time points 10 or 15 weeks after having HFD (Figure S3). Exercise during weeks 0–10 and 5–15 might be able to enhance memory function in APP-HFD mice. These results collectively indicated that consumption of HFD after finishing exercise could disrupt the positive effect of exercise on memory function in APP transgenic mice, but not in WT mice.

### Improvement of Aβ pathology by exercise was abolished by HFD in APP transgenic mice

**Figure 4:**
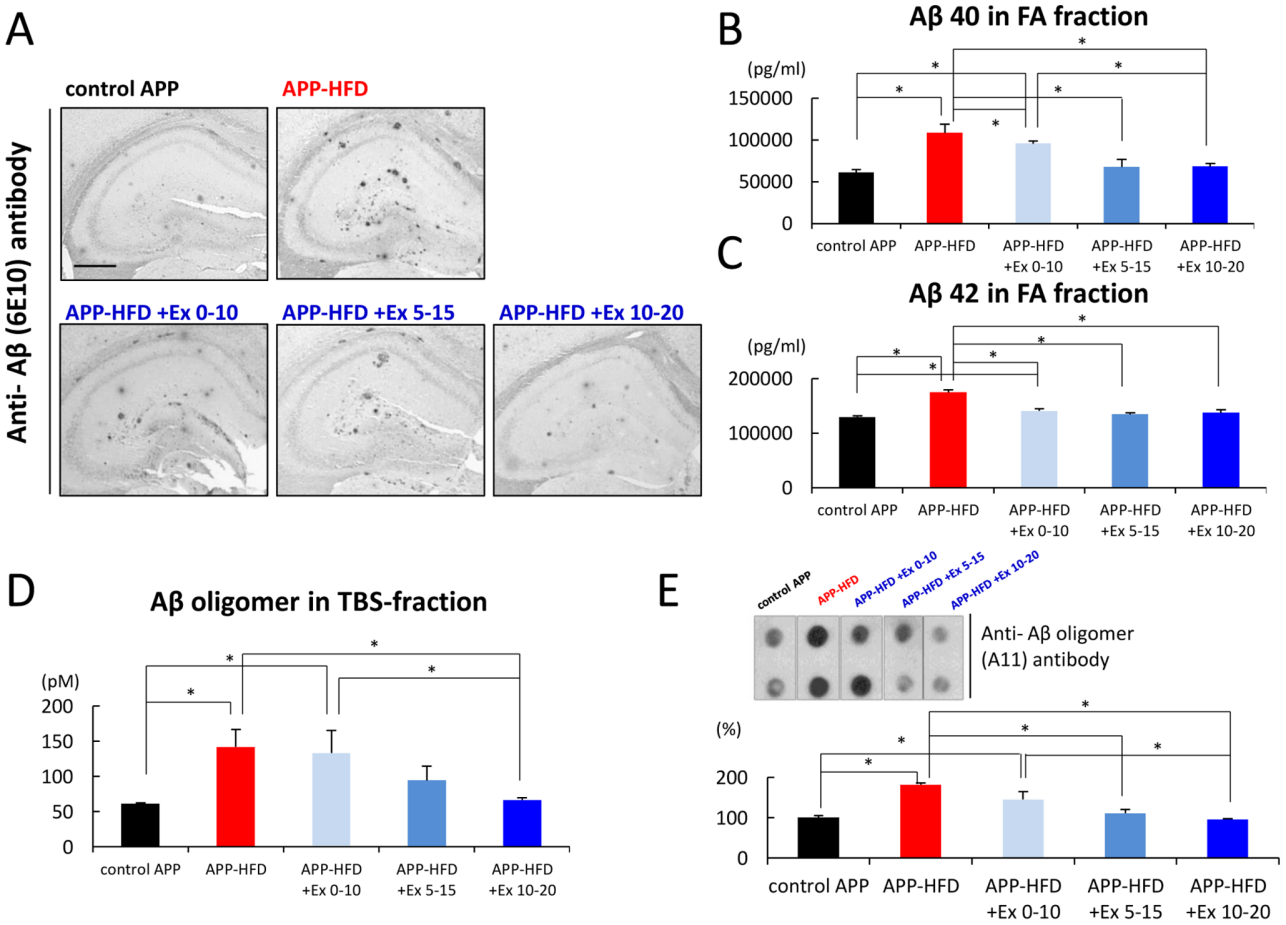
HFD after exercising increased Aβ oligomer as well as deposited Aβ levels in APP-HFD mice. (*A*) Immunohistochemical analysis using anti-Aβ (6E10) antibody. Representative images of Aβ-immunostained hippocampus sections from control APP, APP-HFD, APP-HFD+Ex 0–10, APP-HFD+Ex 5–15 and APP-HFD+Ex 10–20 mice, respectively. Scale bar, 0.5 mm. Increase of Aβ deposition was observed in APP-HFD+Ex 0–10and APP-HFD+Ex 5–15 mice compared with that in APP-HFD+Ex 10–20 mice. However, the amount of Aβ deposition in APP-HFD+Ex 0–10 mice and APP-HFD+Ex 5–15 mice was less than that in APP-HFD mice. (*B*) The amount of Aβ 40 in FA fraction of control APP, APP-HFD, APP-HFD+Ex 0–10, APP-HFD+Ex 5–15 and APP-HFD+Ex 10–20 mice was analyzed by ELISA. Aβ 40 level in FA fraction of APP-HFD+Ex 0–10 mice was higher than that of APP-HFD+Ex 10–20 mice (F _(4, 15)_  = 10.40, p = 0.009). However, the amount of Aβ 40 in APP-HFD+Ex 0–10 mice (p = 0.028) and APP-HFD+Ex 5–15 mice (p = 0.004) was less than that in APP-HFD mice. * indicated p<0.05. (*C*) The amount of Aβ 42 in FA fraction of control APP, APP-HFD, APP-HFD+Ex 0–10, APP-HFD+Ex 5–15 and APP-HFD+Ex 10–20 mice was analyzed by ELISA. There was no statistically significant difference in the level of Aβ 42 among APP-HFD+Ex 0–10, APP-HFD+Ex 5–15 and APP-HFD+Ex 10–20 mice (F _(4, 15)_  = 24.4). However, the amount of Aβ 42 in APP-HFD+Ex 0–10 mice (p<0.001) and APP-HFD+Ex 5–15 mice (p<0.001) was lower than that in APP-HFD mice. * indicated p<0.05. (*D*) The amount of Aβ oligomer in the TBS-soluble fraction of control APP, APP-HFD, APP-HFD+Ex 0–10, APP-HFD+Ex 5–15 and APP-HFD+Ex 10–20 mice was analyzed by ELISA. The level of Aβ oligomer in TBS fraction of APP-HFD+Ex 0–10 mice was higher than that of APP-HFD+Ex 10–20 mice (F _(4, 15)_  = 3.33, p = 0.035). The amount of Aβ oligomer in APP-HFD+Ex 0–10 mice was the same as that in APP-HFD mice. * indicated p<0.05. (*E*) The amount of Aβ oligomer in the TBS-soluble fraction of control APP, APP-HFD, APP-HFD+Ex 0–10, APP-HFD+Ex 5–15 and APP-HFD+Ex 10–20 mice was analyzed by filter trap assay using anti-oligomer (A11) antibody to detect oligomeric Aβ. Representative images of dot are shown in upper panel. Statistical analysis of dot density is described at the bottom. The average dot density of the control APP samples was regarded as 100% and that of other groups was relatively indicated. The relative density of APP-HFD+Ex 0–10 mice was higher than that of APP-HFD+Ex 10–20 mice (F _(4, 10)_  = 12.69, p = 0.007). The dot density of Aβ oligomer in APP-HFD+Ex 0–10 mice was the same as that in APP-HFD mice. * indicated p<0.05.

As shown in our previous report, HFD aggravated the oligomerization and accumulation of Aβ, whereas a marked reduction of the same was observed in APP-HFD+Ex 10–20 mice [10]. Immunohistochemical analysis showed that the accumulated Aβ level in APP-HFD+Ex 0–10 mice was higher than that in APP-HFD+Ex 10–20 mice (Figure 4A). ELISA also showed that the Aβ 40 level in FA fraction of APP-HFD+Ex 0–10 mice was higher than that of APP-HFD+Ex 10–20 mice (Figure 4B). There was no difference between the levels of Aβ 42 in APP-HFD+Ex 0–10 and in APP-HFD+Ex 10–20 mice (Figure 4C). Both Aβ 40 and Aβ 42 levels in APP-HFD+Ex 0–10 were lower than those in APP-HFD mice (Figure 4B and C). TBS-soluble Aβ oligomers are known as the most toxic form of Aβ correlating with memory deficits in AD model mice [18]–[20]. ELISA showed that the level of Aβ oligomer in APP-HFD+Ex 0–10 was significantly higher than that in APP-HFD+Ex 10–20 mice (Figure 4D). Filter trap analysis using anti-oligomer antibody to detect oligomeric Aβ species also showed the same tendency in the amount of Aβ oligomer by ELISA assay (Figure 4E). Interestingly, the level of Aβ oligomer in APP-HFD+Ex 0–10 was the same as that in APP-HFD mice (Figure 4D and E). The amount of Aβ in FA fraction as well as Aβ oligomer in APP-HFD+Ex 5–15 mice were at intermediate levels between those in APP-HFD+Ex 0–10 mice and in APP-HFD+Ex 10–20 mice. These results collectively indicated that HFD after finishing exercise might revive the level of soluble Aβ oligomers.

### APP-CTFβ was accumulated in the APP mice having HFD after finishing exercise

**Figure 5:**
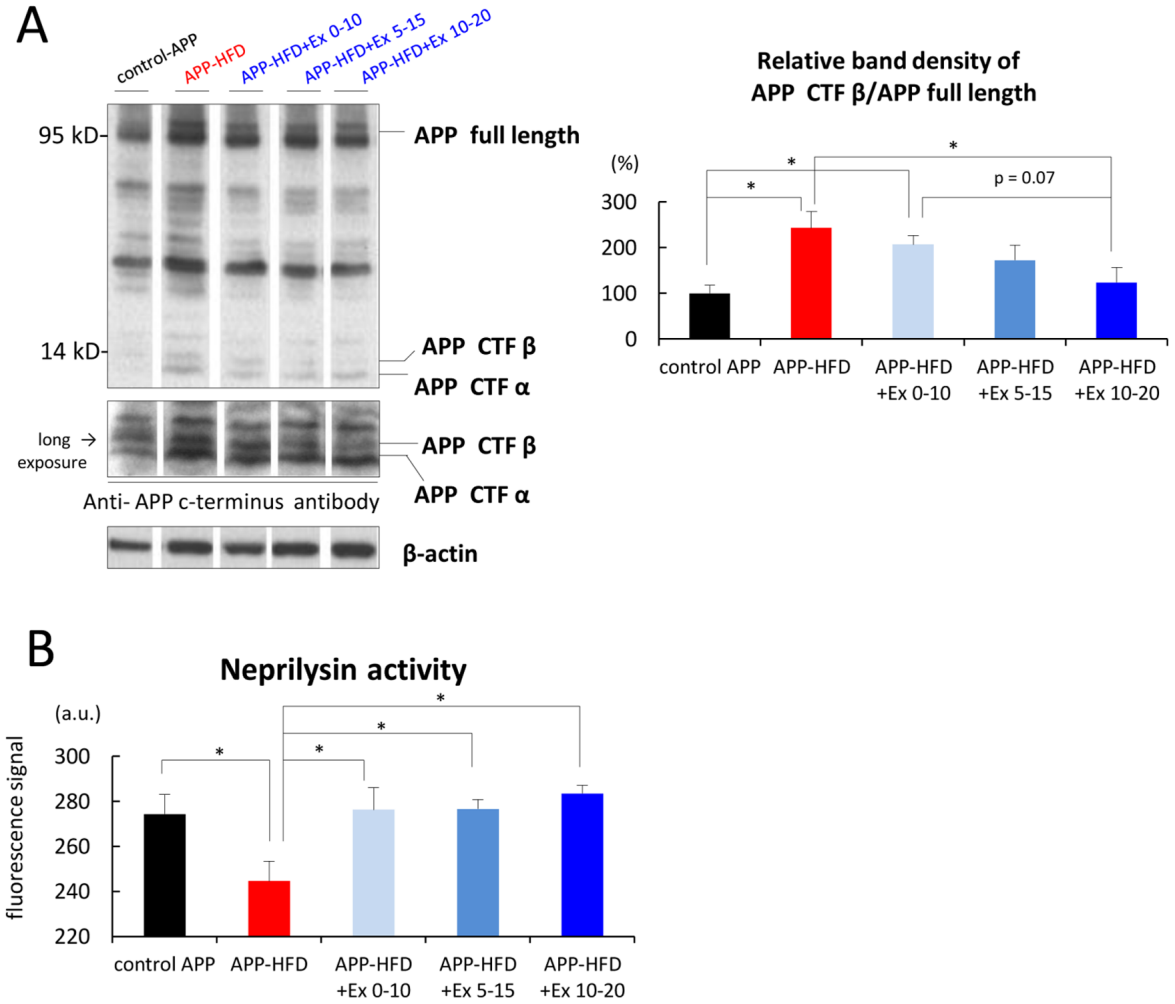
HFD after finishing exercise promoted APP CTFβ accumulation in APP-HFD mice. (*A*) Immunoblotting analysis of APP full length and APP-CTFβ using APP C-terminus antibody. The number of animals used for immunoblotting analysis is 3–4 per group. Statistical analysis is shown in the right panel. The band of APP CTFβ was normalized by that of APP full length. The band density of the control APP mice was regarded as 100% and that of other groups was relatively indicated. The band density of APP CTFβ in APP-HFD+Ex 0–10 mice tended to be higher compared with that in APP-HFD+Ex 10–20 mice (F _(4, 13)_  = 3.70, p = 0.070). * indicated p<0.05. (*B*) *In vitro* enzyme activity assay of neprilysin using fluorescent substrate. The activity of neprilysin in APP-HFD+Ex 0–10 or APP-HFD+Ex 5–15 mice was not different from that in APP-HFD+Ex 10–20 mice (F _(4, 15)_  = 4.10). * indicated p<0.05.

From the above results, we wondered whether the level of Aβ oligomers in our mice was regulated by APP processing or by Aβ degradation. In order to investigate the effect of HFD after exercising on APP processing, we analyzed the level of APP C-terminus fragment (CTF) β through immunoblotting assay. α- and β-secretases cleave APP at the extramembrane domain, producing APP-CTFα and APP-CTFβ respectively. γ-Secretase then cleaves APP-CTFα and APP-CTFβ at the intramembrane domain, producing p3 and Aβ respectively. The level of APP-CTFβ in APP-HFD+Ex 0–10 mice was higher than that in APP-HFD+Ex 10–20 mice, although it did not reach the level where it could be considered statistically significant (Figure 5A). The level of APP-CTFβ in APP-HFD+Ex 5–15 mice was at intermediate level between that in APP-HFD+Ex 0–10 mice and in APP-HFD+Ex 10–20 mice. This result indicated that HFD after finishing exercise might promote the cleavage of APP by β-secretase, followed by the accumulation of APP-CTFβ ˜ Next, we examined the effect of HFD after exercising on Aβ degradation. Since we have previously reported that exercise strengthens the enzymatic activity of neprilysin, an Aβ-degrading protease, in APP-HFD mice [11], we conducted *in vitro* neprilysin activity assay. Notably, the enzymatic activity of neprilysin in APP-HFD+Ex 0–10 mice or in APP-HFD+Ex 5–15 mice was the same as that in APP-HFD+Ex 10–20 mice (Figure 5B). This result indicated that HFD for 10 weeks was not sufficient to abolish the exercise-induced up-regulation of neprilysin activity.

## Discussion

Many reports have focused on the pathological roles of familial genes as a contributory factor in AD and their functions have been clarified gradually. On the other hand, increasing reports have recently examined the effects of environmental factors on AD pathology [21]–[29]. Importantly, epidemiological and experimental reports have shown that metabolic conditions caused by high caloric intake constitute an important risk factor in the development of sporadic AD [21], [22], [25]. Consistent with these backgrounds, it has been demonstrated that feeding HFD to APP mice shows significant deterioration of memory function [7]. It has also been shown that voluntary exercise is effective in the improvement of HFD-induced Aβ deposition and memory deficit [10]. In the present study, we showed the novel finding that the beneficial effect of exercise on memory function could last for a long time in WT mice, whereas it was immediately abolished in APP transgenic mice if they continued to take HFD after finishing exercise.

It has been proposed that hyperglycemia causes damage to neurons due to increase in the reactive oxygen species [30]. Also, HFD may lead to chronic cerebral hypoperfusion, which induces impairment of working memory [31]. HFD may also lead to a breakdown of the blood-brain barrier, resulting in the leakage of serum-derived components into the brain parenchyma, leading to neuronal dysfunction [32]. They might lead to memory impairment in WT-HFD mice. Actually, obesity and glucose intolerance were clearly observed in WT-HFD+Ex 0–10 and WT-HFD+Ex 5–15 mice (Figure 2), indicating that HFD after finishing exercise disrupted metabolic conditions, which might also damage neuronal functions in these mice. However, in the present study, we showed that the beneficial effect of exercise on memory function was maintained in WT-HFD mice even though they stopped exercising (Figure 3). As a mechanism, we estimated that the beneficial roles of exercise might keep counteracting HFD-induced neuronal damages (i.e. from reactive oxygen species) in WT-HFD mice. According to previous reports, exercise enhances neurogenesis and increases the number of synapses [9]. Besides, exercise regulates neuronal development as well as plasticity [33]. Therefore, the pathways up-regulated by exercise might be different from those down-regulated by HFD.

On the other hand, APP-HFD mice show more significant memory impairment than WT-HFD mice [10], suggesting that the memory impairment in APP-HFD mice was attributable to an interaction between HFD and APP metabolism. In the present study, exercise during early periods was able to inhibit HFD-induced memory impairment in APP-HFD mice (Figure S3). However, the beneficial effect of exercise on memory function was immediately abolished in APP-HFD mice when they kept having HFD after exercising (Figure 3). We observed that toxic Aβ oligomer level in APP-HFD+Ex0–10 mice was the same as that in APP-HFD mice. We also observed that the level of deposited Aβ in APP-HFD+Ex0–10 mice was lower than that in APP-HFD mice (Figure 4). Since the degree of memory impairment in APP-HFD+Ex0–10 mice was the same as that in APP-HFD mice, we speculated that the increase of soluble Aβ oligomer by HFD after finishing exercise might be sufficient to lead to memory loss. Then we examined the molecular mechanisms, based on which HFD after exercising increased Aβ oligomer level. In our previous report, we demonstrated that HFD may promote the cleavage of APP by β-secretase leading to the production of Aβ, but exercise inhibited it. As a mechanism, we have demonstrated that HFD leads to metabolic conditions such as obesity and glucose abnormalities, followed by up-regulating β-secretase enzyme activity. But exercise can down-regulate β-secretase enzyme activity since it improves HFD-induced metabolic condition [10]. In the present study, we showed that HFD might increase the level of APP-CTFβ after finishing exercise (Figure 5). Thus, once exercise is finished, HFD might rapidly re-increase APP processing. In this sense, the effect of exercise on the inhibition of β-secretase activity might be temporary. We had recently shown that exercise strengthens the enzymatic activity of neprilysin, which may promote the degradation of Aβ [11]. However, in the present study, we showed that HFD after exercising did not lower neprilysin activity (Figure 5). According to these results, we speculated that HFD-promoted Aβ production might be the reason why HFD after finishing exercise increased Aβ oligomer as well as deposited Aβ. However, previous literature has reported that HFD suppresses the activity of insulin-degrading enzyme [7]. Thus, the effects of another Aβ-degrading enzyme on our results should be clarified in future studies.

Verret et al. have reported that in WT mice, an enriched environment during earlier period has the same effect on memory function as later one under a standard diet condition [34]. Although there is a discrepancy between their experimental setting and ours, we consistently showed that the effect of exercise on memory was maintained even under a HFD condition in WT mice (Figure 3). Verret et al. have also shown that an enriched environment during earlier period is more effective in reserving memory function than that during later period in APP transgenic mice having standard diet [34]. However, we showed that the effect of exercise during earlier period on memory function was clearly reduced 20 weeks after having HFD (Figure 3). In this sense, whether the effect of exercise is maintained might depend on the metabolic conditions in APP transgenic mice. However, there was a limitation in the experiment using APP transgenic mice. In this report, we showed that only 10 weeks of HFD after finishing exercise was sufficient to increase Aβ oligomer level and subsequently, to disrupt exercise-protected memory function. This rapid alteration might be due to the experimental strategy using transgenic model mice overexpressing APP and producing excess amount of Aβ. In fact, a previous study using *in vivo* multiphoton microscopy has reported that Aβ plaques form extraordinarily quickly, over 24 hours, in model mice of AD [35]. Such a rapid alteration of Aβ deposition may not be relevant in sporadic human AD cases. Considering this discrepancy between APP transgenic mice and human AD cases, it is not clear whether HFD abolishes the beneficial effect of exercise on memory function in human cases as rapidly as in APP transgenic mice. Nevertheless, our findings clearly indicate that continuation of exercise is necessary to rescue HFD-induced aggravation of cognitive decline in APP transgenic mice. Given that the effect of exercise depends on metabolic conditions, one's dietary pattern should be considered a very important factor in the prevention of AD.

## Supporting Information

Figure S1 **Every week monitoring of the amount of food intake.** Every week monitoring showed that average amount of food intake in control APP, APP-HFD, APP-HFD+Ex 0–10, APP-HFD+Ex 5–15 and APP-HFD+Ex 10–20 mice. During the induction of exercise, APP-HFD+Ex 0–10, APP-HFD+Ex 5–15 and APP-HFD+Ex 10–20 mice tended to take more food than APP-HFD mice did.
(PDF)

Figure S2 **Swimming speeds in Morris water maze test.** Locomotor activities of control APP, APP-HFD, APP-HFD+Ex 0–10, APP-HFD+Ex 5–15 and APP-HFD+Ex 10–20 mice were analyzed by swimming speeds in the visual cue phase of Morris water maze tests 10, 15 (A) and 20 weeks (B) after having HFD. There were no statistical differences among control APP, APP-HFD, APP-HFD+Ex 0–10, APP-HFD+Ex 5–15 and APP-HFD+Ex 10–20 mice.
(PDF)

Figure S3 **Exercise at different periods were able to strengthen memory function in APP-HFD mice.** 10 weeks after having HFD, the acquisition time was clearly shortened in APP-HFD+Ex 0–10 mice (Figure S3B, left). Furthermore, 15 weeks after having HFD, the acquisition time was also ameliorated in APP-HFD+Ex 5–15 mice (Figure S3B, right). These results indicated that exercise during weeks 0–10 and weeks 5–15 could strengthen memory function in APP-HFD mice. However, at 10 weeks after having HFD, the improvement in the acquisition time was not observed in APP-HFD+Ex 10–20 mice, indicating that HFD for 10 weeks was sufficient to induce memory loss in APP transgenic mice (Figure S3B, left). We also conducted the pilot study in WT-HFD mice using the same strategy in APP-HFD mice. Although HFD for 10 weeks was sufficient to lead to memory deficit in APP transgenic mice, HFD for 10 weeks did not induce memory impairment in WT mice (Figure S3C). (*A*) Schematic presentation of the pilot study in Morris water maze test. Morris water maze test was conducted 10 weeks and 15 weeks after having HFD. (*B*) The time to get to the goal platform of exercise-treated APP-HFD mice in the acquisition phase, 10 weeks (left) and 15 weeks (right) after having HFD. 10 weeks after having HFD, APP transgenic mice having HFD (APP-HFD+Ex 10–20 mice) took significant longer time to the platform. On the other hand, APP-HFD+Ex 0–10 mice clearly took shorter time to the platform than APP-HFD+Ex 10–20 mice. (*C*) The time to get to the goal platform of exercise-treated WT-HFD mice in the acquisition phase, 10 weeks (left) and 15 weeks (right) after having HFD. 10 weeks after having HFD, WT mice having HFD (WT-HFD+Ex 10–20 mice) did not take longer time to the platform. WT-HFD+Ex 0–10 mice took the same time to the platform as WT-HFD+Ex 10–20 mice.
(PDF)
