# Supplementary figures and images for: Continuation of Exercise Is Necessary to Inhibit High Fat Diet-Induced β-Amyloid Deposition and Memory Deficit in Amyloid Precursor Protein Transgenic Mice
Source: PLoS One. 2013 Sep 4;8(9):e72796. doi: 10.1371/journal.pone.0072796 (PMC3762856; doi:10.1371/journal.pone.0072796)

**Figure. S1.** Every week monitoring of the amount of food intake

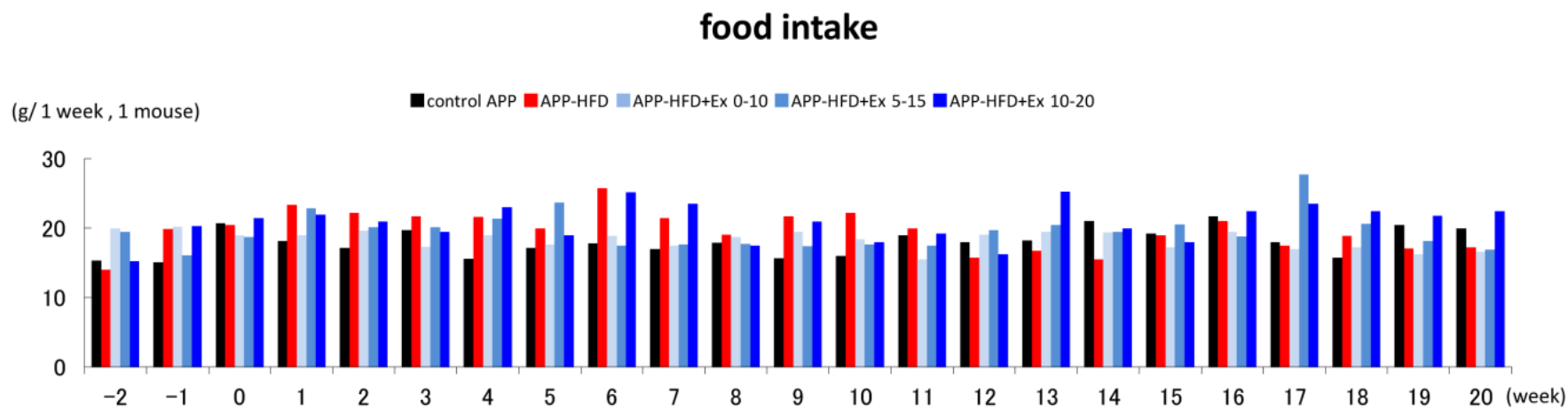

Supplement: Figure S1 — Every week monitoring of the amount of food intake. Every week monitoring showed that average amount of food intake in control APP, APP-HFD, APP-HFD+Ex 0–10, APP-HFD+Ex 5–15 and APP-HFD+Ex 10–20 mice. During the induction of exercise, APP-HFD+Ex 0–10, APP-HFD+Ex 5–15 and APP-HFD+Ex 10–20 mice tended to take more food than APP-HFD mice did. (PDF) [file pone.0072796.s001.pdf]

**Figure. S2.** Swimming speeds in Morris water maze test

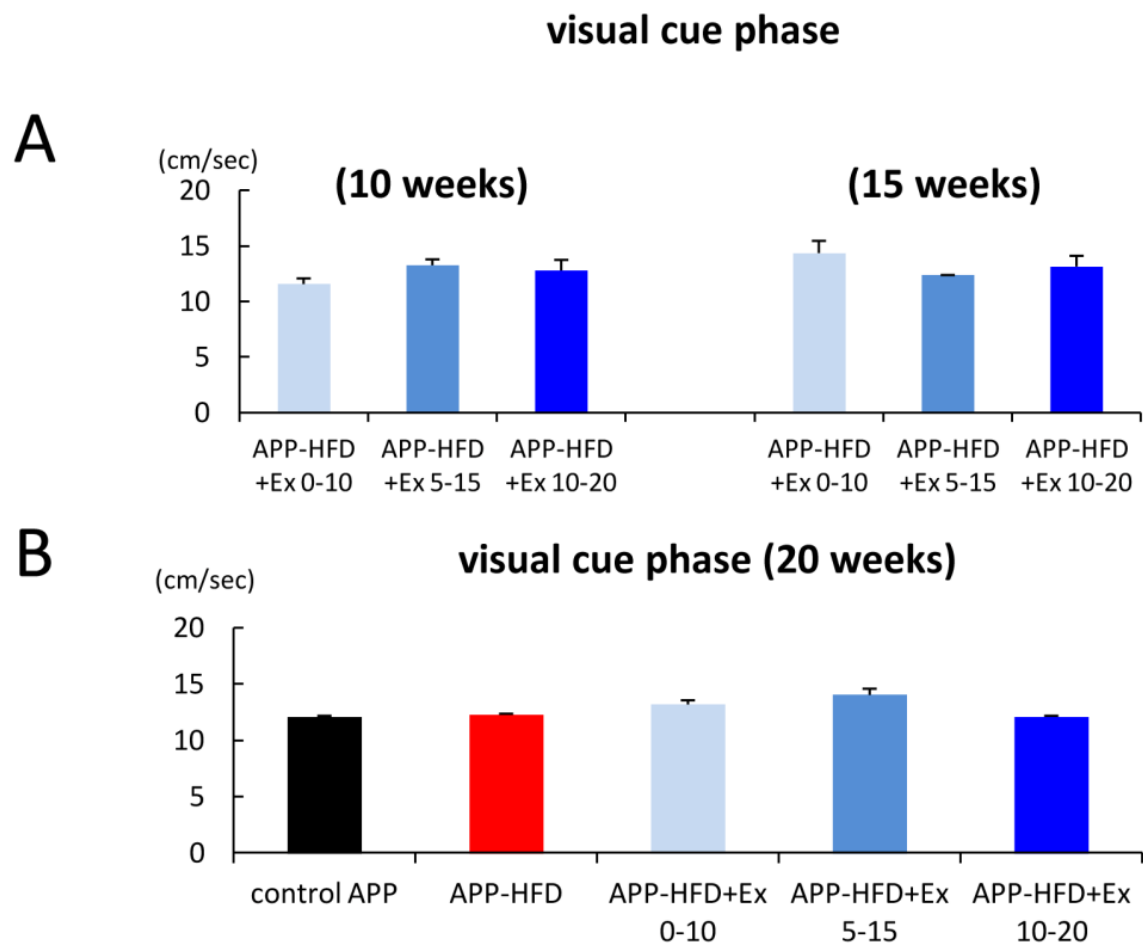

Supplement: Figure S2 — Swimming speeds in Morris water maze test. Locomotor activities of control APP, APP-HFD, APP-HFD+Ex 0–10, APP-HFD+Ex 5–15 and APP-HFD+Ex 10–20 mice were analyzed by swimming speeds in the visual cue phase of Morris water maze tests 10, 15 (A) and 20 weeks (B) after having HFD. There were no statistical differences among control APP, APP-HFD, APP-HFD+Ex 0–10, APP-HFD+Ex 5–15 and APP-HFD+Ex 10–20 mice. (PDF) [file pone.0072796.s002.pdf]

**Figure. S3.** Exercise at different periods were able to strengthen memory function in APP-HFD mice

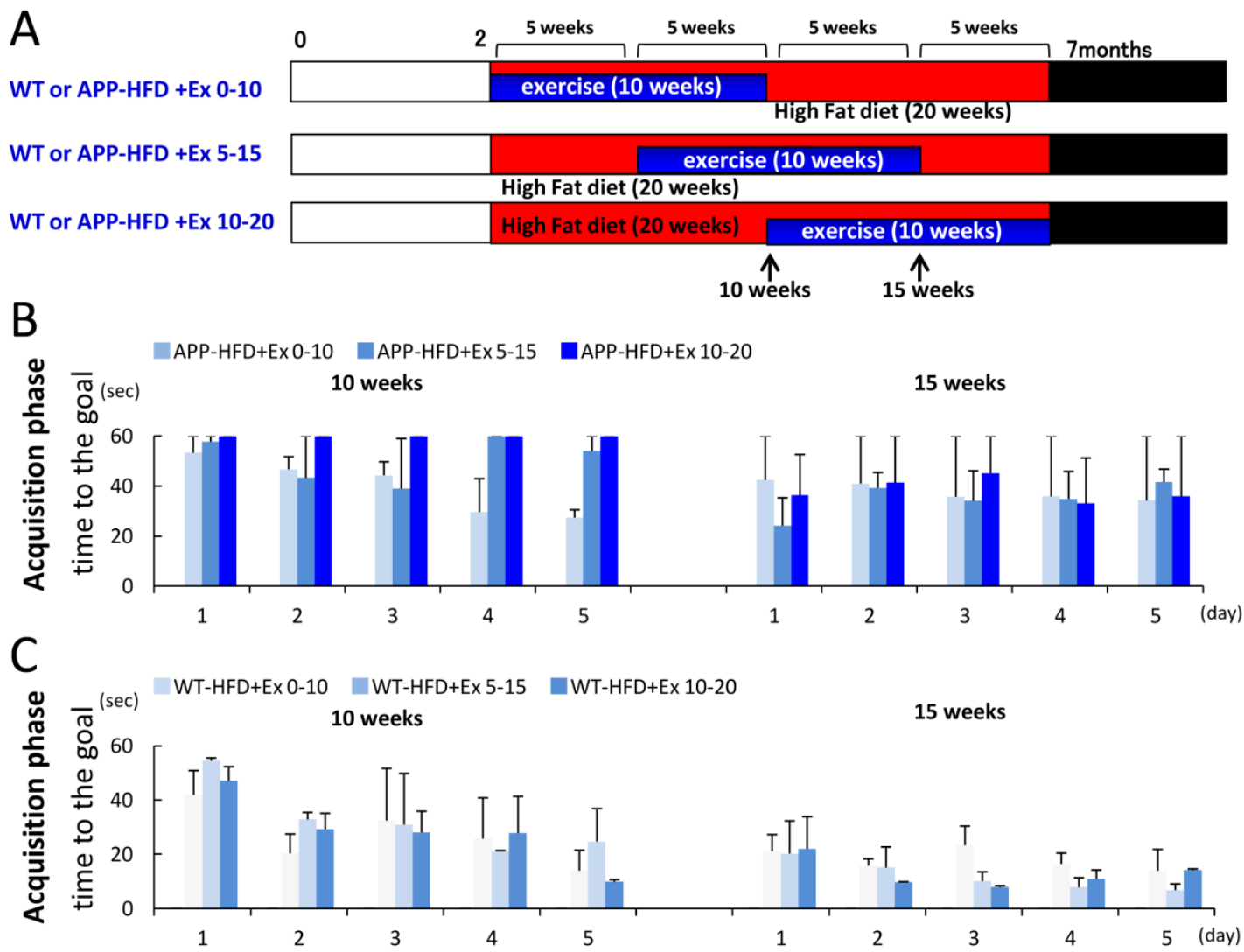

Supplement: Figure S3 — Exercise at different periods were able to strengthen memory function in APP-HFD mice. 10 weeks after having HFD, the acquisition time was clearly shortened in APP-HFD+Ex 0–10 mice (Figure S3B, left). Furthermore, 15 weeks after having HFD, the acquisition time was also ameliorated in APP-HFD+Ex 5–15 mice (Figure S3B, right). These results indicated that exercise during weeks 0–10 and weeks 5–15 could strengthen memory function in APP-HFD mice. However, at 10 weeks after having HFD, the improvement in the acquisition time was not observed in APP-HFD+Ex 10–20 mice, indicating that HFD for 10 weeks was sufficient to induce memory loss in APP transgenic mice (Figure S3B, left). We also conducted the pilot study in WT-HFD mice using the same strategy in APP-HFD mice. Although HFD for 10 weeks was sufficient to lead to memory deficit in APP transgenic mice, HFD for 10 weeks did not induce memory impairment in WT mice (Figure S3C). (A) Schematic presentation of the pilot study in Morris water maze test. Morris water maze test was conducted 10 weeks and 15 weeks after having HFD. (B) The time to get to the goal platform of exercise-treated APP-HFD mice in the acquisition phase, 10 weeks (left) and 15 weeks (right) after having HFD. 10 weeks after having HFD, APP transgenic mice having HFD (APP-HFD+Ex 10–20 mice) took significant longer time to the platform. On the other hand, APP-HFD+Ex 0–10 mice clearly took shorter time to the platform than APP-HFD+Ex 10–20 mice. (C) The time to get to the goal platform of exercise-treated WT-HFD mice in the acquisition phase, 10 weeks (left) and 15 weeks (right) after having HFD. 10 weeks after having HFD, WT mice having HFD (WT-HFD+Ex 10–20 mice) did not take longer time to the platform. WT-HFD+Ex 0–10 mice took the same time to the platform as WT-HFD+Ex 10–20 mice. (PDF) [file pone.0072796.s003.pdf]
